# NOX2-Driven Oxidative Stress and Endothelial Dysfunction in Male Elite Adolescent Soccer Players

**DOI:** 10.3390/biom16091301

**Published:** 2026-09-09

**Authors:** Chiara Fossati, Federica Armeli, Alessandra D’Amico, Giacomo Frati, Giovanni Enrico Casciaro, Elena Cavarretta, Vittoria Testa, Alessio Borrelli, Luigi Sciarra, Chiara Lodoli, Marco Zagarella, Beatrice Oana Paun, Vittorio Picchio, Antonio Gianfelici, Fabio Pigozzi, Stefano Corrado, Fabrizio Perroni, Cristina Nocella, Roberto Carnevale

**Affiliations:** 1 Department of Movement, Human and Health Sciences, University of Rome “Foro Italico”, 00135 Rome, Italy; chiara.fossati@uniroma4.it (C.F.); a.gianfelici@studenti.uniroma4.it (A.G.); fabio.pigozzi@uniroma4.it (F.P.); 2 Department of Medical and Surgical Sciences and Biotechnologies, Sapienza University of Rome, 04100 Latina, Italy; federica.armeli@uniroma1.it (F.A.); giacomo.frati@uniroma1.it (G.F.); giovannienrico.casciaro@uniroma1.it (G.E.C.); elena.cavarretta@uniroma1.it (E.C.); vittorio.picchio@uniroma1.it (V.P.); 3 Department of Health and Life Sciences, European University of Rome, 00163 Rome, Italy; alessandra.damico@unier.it; 4 IRCCS NeuroMed, 86077 Pozzilli, Italy; 5 Advanced Cardiovascular Therapies Unit, Bambino Gesù Children’s Hospital, IRCCS, 00165 Rome, Italy; 6 Corso di Laurea di I Livello in Infermieristica, Sapienza University of Rome Polo Pontino-Sede di Terracina, 04019 Terracina, Italy; testa.2127474@studenti.uniroma1.it; 7 Department of Life, Health and Environmental Sciences, University of L’Aquila, 67100 L’Aquila, Italy; alessioborrelli@libero.it (A.B.); luigi.sciarra@univaq.it (L.S.); 8 Department of Maternal and Child Health and Urological Sciences—Policlinico Umberto I, 00161 Rome, Italy; chiara.lodoli@uniroma1.it; 9 Faculty of Medicine and Surgery, Sapienza University of Rome, 04100 Latina, Italy; zagarella.1939512@studenti.uniroma1.it (M.Z.); paun.2146395@studenti.uniroma1.it (B.O.P.); 10 Department of Human Sciences, Society and Health, University of Cassino and Southern Lazio, 03043 Cassino, Italy; corrado.stefano87@gmail.com; 11 Department of Biomolecular Sciences, Section of Exercise and Health Sciences, University of Urbino Carlo Bo, 61029 Urbino, Italy; fabrizio.perroni@uniurb.it; 12 Istituto Pasteur Italia—Fondazione Cenci Bolognetti, 00161 Roma, Italy; cristina.nocella@uniroma1.it; 13 Department of Medical and Cardiovascular Sciences, Sapienza University of Rome, 00161 Rome, Italy

**Keywords:** NOX2, oxidative stress, endothelial impairment, salivary biomarkers, adolescent elite athletes

## Abstract

Background: Intense physical exercise induces oxidative stress and inflammation in adult professional athletes, but data on adolescent elite athletes remain limited. Methods: Twenty-four elite adolescent soccer players were recruited and evaluated three times: pre-season baseline (T0), after one month of pre-season training (T1), and at the end of the first half of the competitive season (T2, approximately 4 months after T0). Saliva samples were collected as non-invasive surrogate indicators reflecting oxidative stress- and endothelial-related signaling. In vitro studies were performed on endothelial cells exposed to H_2_O_2_ concentrations observed in athletes. Results: Athletes showed a significant increase in salivary oxidative stress markers (NOX2 levels and H_2_O_2_) at T1 and T2 versus T0. Endothelial impairment was evidenced by reduced salivary NOx concentration and elevated endothelin (ET-1) levels. In vitro, endothelial cells exposed to H_2_O_2_ (8 μM) showed increased sNOX2-dp and ET-1 levels, reduced eNOS phosphorylation, and impaired tube formation (mesh number and area) compared with untreated cells. NOX2ds-tat treatment reduced cell damage and restored angiogenic capacity. Conclusions: Our findings indicate that adolescent elite athletes experience significant salivary oxidative stress- and endothelial-related alterations, potentially reflecting cardiovascular stress. These results underscore the importance of cardiovascular monitoring and the potential benefits of antioxidant strategies, even in young athletes participating in high-intensity sports.

## 1. Introduction

Regular physical activity is widely recognized as a key determinant of cardiovascular health. It is associated with improved endothelial function through both hemodynamic and molecular adaptations [1,2], reducing cardiovascular risk, and enhanced metabolic regulation [3]. When performed regularly, moderate exercise promotes physiological adaptations that improve vascular homeostasis and nitric oxide bioavailability, thereby supporting cardiovascular protection [4]. It is well established that reactive oxygen species (ROS) play a fundamental physiological role in regulating normal vascular function, arterial tone, stress adaptation, and physiological remodeling [5]. However, while physiological ROS levels act as crucial signaling molecules, strenuous or exhaustive exercise may induce an excessive increase in ROS production, driven by activation of redox-dependent signaling pathways involved in both exercise adaptation and acute oxidative imbalance [6]. Exercise-induced ROS production originates from multiple cellular sources, including mitochondria and enzymatic sources such as xanthine oxidase and NADPH oxidases. Among these, NADPH oxidase isoform 2 (NOX2) has been extensively investigated in the cardiovascular field and implicated in mechanisms underlying vascular dysfunction [7,8]. Clinical evidence indicates that increased NOX2 levels are associated with arterial dysfunction and elevated systemic oxidative stress in humans [9]. Moreover, NOX2-derived ROS play an important role in redox signaling during physical activity and contribute to the molecular responses induced by acute exercise. Several studies support the role of NOX2 as a major source of ROS generation during exercise. In mice carrying a loss-of-function mutation in the regulatory NOX2 subunit p47phox, ROS production was completely abolished following an acute bout of moderate-intensity treadmill exercise [10]. Furthermore, our group previously reported that elite athletes engaged in highly intensive sports exhibit significantly higher NOX2 levels compared with amateur athletes, accompanied by a marked reduction in antioxidant capacity activation [11]. In addition to oxidative stress, intense physical activity can stimulate a systemic inflammatory response [12]. This temporary response is characterized by increased levels of inflammatory cytokines, particularly interleukin-6 (IL-6) and C-reactive protein (CRP) [12,13]. These processes may amplify ROS production and contribute to tissue damage following strenuous exercise. Although the inflammatory response and redox modulation in the post-exercise period have been extensively described in healthy adult athletes [14,15], the effects of intense exercise on redox homeostasis in adolescents remain poorly investigated. This population represents a unique physiological context characterized by rapid, ongoing physical and neuromuscular growth, accompanied by hormonal changes and cardiovascular maturation [16]. Furthermore, early ontogenesis and juvenile development represent stages with heightened baseline ROS-dependent signaling, where vascular remodeling and cellular proliferation are particularly active [17]. Interestingly, previous research has shown that NOX2-derived oxidative stress is already detectable during early life and may contribute to oxidative processes in childhood [18]. Furthermore, increased NOX2 activity in pediatric populations has been associated with markers of endothelial dysfunction and vascular impairment [18], suggesting that oxidative stress may influence cardiovascular health even at young ages. Whether the heightened NOX2 activity observed in young athletes stems from developmental baseline characteristics or reflects exercise-induced oxidative load remains an open question. The increasing participation of adolescents in competitive sports raises important questions regarding the potential cardiovascular impact of intense training loads during this critical developmental period. Indeed, the impact of training during this critical developmental period is particularly important, as it may not only affect immediate performance but also contribute to long-term motor adaptations. A systematic review has reported the release of cardiac troponin in adolescent swimmers following exertion [19]. Similarly, other studies described the kinetics of high-sensitivity cardiac troponin T (hs-cTnT) after prolonged exercise in adolescents and adults, demonstrating a significantly greater post-exercise increase in adolescents than in adults, followed by subsequent normalization [20,21]. These elevations are not indicative of irreversible myocardial necrosis but may reflect increased cardiac cellular stress and increased transient membrane permeability [20,21]. Among potential mechanisms underlying these responses, endothelial activation and redox imbalance have been proposed as contributors to transient cardiovascular alterations in young athletes. Saliva has emerged as a valuable diagnostic biofluid, as the composition of saliva dynamically reflects physiological and pathological changes occurring throughout the body, making it an attractive and non-invasive source of clinically relevant biomarkers. Salivary redox biomarkers have been increasingly investigated for the diagnosis and monitoring of a wide range of disorders, including metabolic diseases such as obesity, insulin resistance, diabetes mellitus, and chronic kidney disease, and neurodegenerative disorders, as well as several types of cancer [22]. Non-invasive biomarkers, particularly salivary markers of hydrogen peroxide (H_2_O_2_) [22,23,24,25] and nitric oxide (NO) metabolism [26,27,28], offer a practical, non-invasive surrogate approach reflecting redox- and endothelial-related signaling.

Therefore, the present study aimed to investigate whether sustained high-intensity exercise in adolescent athletes is associated with changes in salivary oxidative stress- and endothelial-related biomarkers, with a particular focus on NOX2-related oxidative stress pathways and salivary biomarkers.

## 2. Materials and Methods

### 2.1. Study Design

Twenty-four male adolescent soccer players (mean age of 15 years) were recruited for this observational study after collection of their medical history and eligibility assessment. Salivary samples were obtained non-invasively at three different standardized time points during the season: pre-season baseline (T0), after one month of pre-season high-volume training (T1), and at the end of the first half of the competitive season (T2; approximately 4 months after T0). The study was conducted according to the ethical principles outlined in the Declaration of Helsinki and approved by the University Committee for Research of the University of Rome “Foro Italico” (Protocol No. CAR 206/2024).

Participants were instructed to avoid antioxidant supplements for one week before saliva sampling; otherwise, no dietary restrictions were imposed, and athletes maintained their usual free diet throughout the study period.

Systolic and diastolic blood pressure were measured at baseline (T0) after a 10 min rest in a seated position using a standardized automated sphygmomanometer with an appropriately sized cuff placed on the dominant arm. Three consecutive measurements were performed at 2 min intervals, and the average of the last two measurements was used for analysis.

### 2.2. Saliva Sample Collection

Saliva specimens were collected by the athletes between 8:00 and 9:00 a.m. to minimize the effect of circadian rhythm on secretion. After rinsing the mouth three times with distilled water at room temperature, the athletes spat out the saliva accumulated at the bottom of the mouth into a sterile Falcon-type tube until approximately 5 mL. Samples were immediately diluted (1:1 *v*/*v*) with sterile phosphate-buffered saline (PBS 1X) to improve handling and pipetting procedures, aliquoted, and stored at −20 °C until analysis. To minimize potential confounding factors, participants were instructed to refrain from consuming food or beverages (except water) and from performing oral hygiene procedures for at least 2 h before saliva collection. Furthermore, no medications were taken within the 8 h preceding sample collection.

Salivary oxidative stress markers serve as non-invasive surrogate indicators reflecting local and systemic redox signaling [29].

### 2.3. Salivary NOX2 Levels

Salivary NOX2 concentration was assessed using a commercial ELISA kit (MyBioSource, San Diego, CA, USA) in duplicate. Standards and samples were incubated in 96-well plates together with HRP-conjugated reagent for 1 h at 37 °C. After the addition of chromogenic substrates, absorbance was measured at 450 nm using a microplate reader. Data were expressed as AU/mL. Intra- and inter-assay coefficients of variation (CV) were both below 15%.

### 2.4. Salivary $H_{2}O_{2}$ Levels

Hydrogen Peroxide (H_2_O_2_) concentrations were evaluated in duplicate through a colorimetric assay kit (Abcam, Cambridge, UK). Quantification was performed using a standard calibration curve (ranging from 0 to 200 µM) of H_2_O_2_.

The assay is based on the Fe^3+^–xylenol orange reaction, in which oxidation of Fe^2+^ by peroxides generates a colored complex proportional to the peroxide concentration. Optical density was recorded at 450 nm, and results were expressed as µM.

### 2.5. Salivary Nitric Oxide (NO)

Nitric oxide production was indirectly evaluated by measuring nitrite/nitrate (NOx) levels in saliva samples in duplicate using a commercial colorimetric assay kit (Abcam, Cambridge, UK). Results are expressed as µM. Intra-assay and inter-assay coefficients of variation were both less than 5%.

### 2.6. Salivary Endothelin-1 Levels

Endothelin-1 (ET-1) concentrations in saliva were quantified in duplicate using a commercial ELISA kit (Elabscience, Wuhan, China). Results are reported as pg/mL. Both intra-assay and inter-assay coefficients of variation were lower than 10%.

### 2.7. Cell Culture and Reagents

Human umbilical vein endothelial cells (HUVECs; Lonza, Basel, Switzerland) were maintained in complete EGM-2 medium (Lonza), supplemented with 10% FBS. Cells, between passages 2 and 3, were seeded at a density of 4000 cells/cm^2^ and cultured for 24 h in a humidified incubator at 37 °C with 5% CO_2_ to sub-confluence levels. Before stimulation, cells were pre-treated for 30 min at 37 °C with the NOX2 inhibitor NOX2ds-tat (10 μM; Anaspec, Fremont, CA, USA). Subsequently, HUVECs were exposed to H_2_O_2_ (2 or 8 µM) for 3 h.

Conditioned media were collected for the determination of H_2_O_2_, sNOX2dp, endothelin-1 levels, and NOx. For protein extraction, cells were lysed in ice-cold RIPA buffer supplemented with protease and phosphatase inhibitors (10 μg/mL; Thermo Fisher Scientific, Waltham, MA, USA).

Lysates were incubated on ice for 30 min and centrifuged at 10,000× *g* for 20 min. Supernatants were collected and used for Western blot analysis of p-eNOS/eNOS expression.

### 2.8. Protein Detection, Electrophoresis, and Western Blot Analysis

Protein concentrations were determined using the DC protein assay (Bio-Rad, Hercules, CA, USA). Equal amounts of protein (30 µg per lane) were mixed with 4× Laemmli buffer containing 20% β-mercaptoethanol, heated at 95 °C for 4 min, and separated by SDS-PAGE on precast polyacrylamide gels.

Proteins were subsequently transferred onto nitrocellulose membranes using the Trans-Blot Turbo system (Bio-Rad, Hercules, CA, USA).

After blocking nonspecific binding sites with EveryBlot Blocking Buffer (Bio-Rad), membranes were incubated overnight at 4 °C with the following antibodies: anti-phospho-eNOS (Ser1177), anti-eNOS (Cell Signaling, Danvers, MA, USA), and anti-actin (Santa Cruz Biotechnology, Dalla, TX, USA). HRP-conjugated secondary antibodies (1:3000 dilution) were incubated for 1 h at room temperature. Protein bands were visualized by enhanced chemiluminescence (ECL; Bio-Rad) and analyzed densitometrically using Image Lab software version 6.1.0. Data are expressed as arbitrary units (A.U.) and represent the mean of three independent experiments.

### 2.9. sNOX2dp Evaluation

NOX2 levels were measured in conditioned media cultures through an ELISA test in duplicate. This assay measures the levels of soluble NOX2-derived peptide (sNOX2-dp) released into the serum following NOX2 activation. Briefly, the procedure included the following steps: (1) coating ELISA 96-well plates overnight at 4 °C with reference standards and serum samples containing 1 µg of protein with known concentrations of sNOX2-dp; (2) washing the wells to remove unbound material; (3) adding horseradish peroxidase (HRP)-conjugated monoclonal anti-sNOX2-dp antibodies directed against the amino acid sequence 224–268 of the extracellular membrane portion of NOX2; and (4) quantifying the bound antibody–enzyme complexes by measuring HRP activity in the presence of the substrate 3,3′,5,5′-tetramethylbenzidine (TMB). Enzymatic activity was determined spectrophotometrically by measuring the increase in absorbance at 450 nm after stopping the reaction with 2 M sulfuric acid. Since absorbance is directly proportional to the concentration of sNOX2-dp, sample values were calculated by interpolation from a standard curve generated using reference standards ranging from 0 to 200 pg/mL in the same assay. Results are expressed as pg/mL. The intra-assay and inter-assay coefficients of variation were 8.95% and 9.01%, respectively.

### 2.10. Angiogenesis

The tube-forming assay was performed on Matrigel-coated 96-well plates with HUVECs (1.5 × 104 cells/well) treated for 16 h with Growth Factor Reduced Matrigel Matrix Phenol Red Free (356234, BD) in the presence of the EGM2 supplemented with 10 μM NOX2ds-tat in the presence or absence of H_2_O_2_. An automated scan of each well was acquired with a 4× objective by a Nikon Eclipse TI inverted microscope with a motorized stage (Nikon Corporation, Tokyo, Japan). Mesh number and area were quantified using ImageJ (v. 1.53k, U. S. National Institutes of Health, Bethesda, MD, USA) with the Angiogenesis plugin.

### 2.11. Statistical Analysis

Baseline clinical characteristics were summarized using descriptive statistics. Categorical variables are presented as absolute frequencies. Continuous variables are reported as mean ± standard deviation (SD) for normally distributed data or as median with interquartile range (IQR) for non-normally distributed data, as appropriate. Normality of distribution was assessed using the Shapiro–Wilk test. For normally distributed variables, repeated-measures analysis of variance (ANOVA) was applied, followed by Bonferroni’s post hoc correction for multiple comparisons. For non-normally distributed variables, the Friedman test was used, followed by Dunn’s post hoc test where appropriate.

In vitro experimental data are presented as mean ± standard deviation (SD) and represent at least three independent experiments. Comparisons between multiple groups were performed using one-way or two-way ANOVA. All statistical tests were two-sided, and a *p*-value < 0.05 was considered statistically significant. Statistical analyses were performed using GraphPad Prism (v. 8.0.2; GraphPad Software, La Jolla, CA, USA).

## 3. Results

### 3.1. In Vivo Study

Table 1 summarizes the clinical and anthropometric characteristics of the study participants.

The sample comprised 24 young, healthy, and physically active males, with a mean age of 15 years. Cardiovascular parameters were within normal ranges. Regarding physical activity, training volume was approximately 12 h per week at the first time point (T1) and approximately 9 h per week at the second time point (T2).

During the pre-season period (T1), elite adolescent soccer players typically followed a structured, high-volume training program aimed at developing general and sport-specific fitness. Sessions included aerobic endurance training (e.g., continuous running and small-sided games with reduced recovery), high-intensity interval training, repeated sprint ability drills, and neuromuscular and strength training (e.g., resistance exercises targeting major muscle groups), as well as technical–tactical drills focusing on ball control, passing, and positional play. Friendly matches were also included to progressively reintroduce competitive demands.

During the competitive season (T2), training load was adjusted to match schedule demands and primarily focused on performance maintenance and recovery. Weekly microcycles generally included tactical and technical sessions with higher specificity, one to two high-intensity field sessions, structured strength maintenance sessions, and one official match per week, with additional recovery or low-intensity regenerative activities following matches.

**Figure 1:**
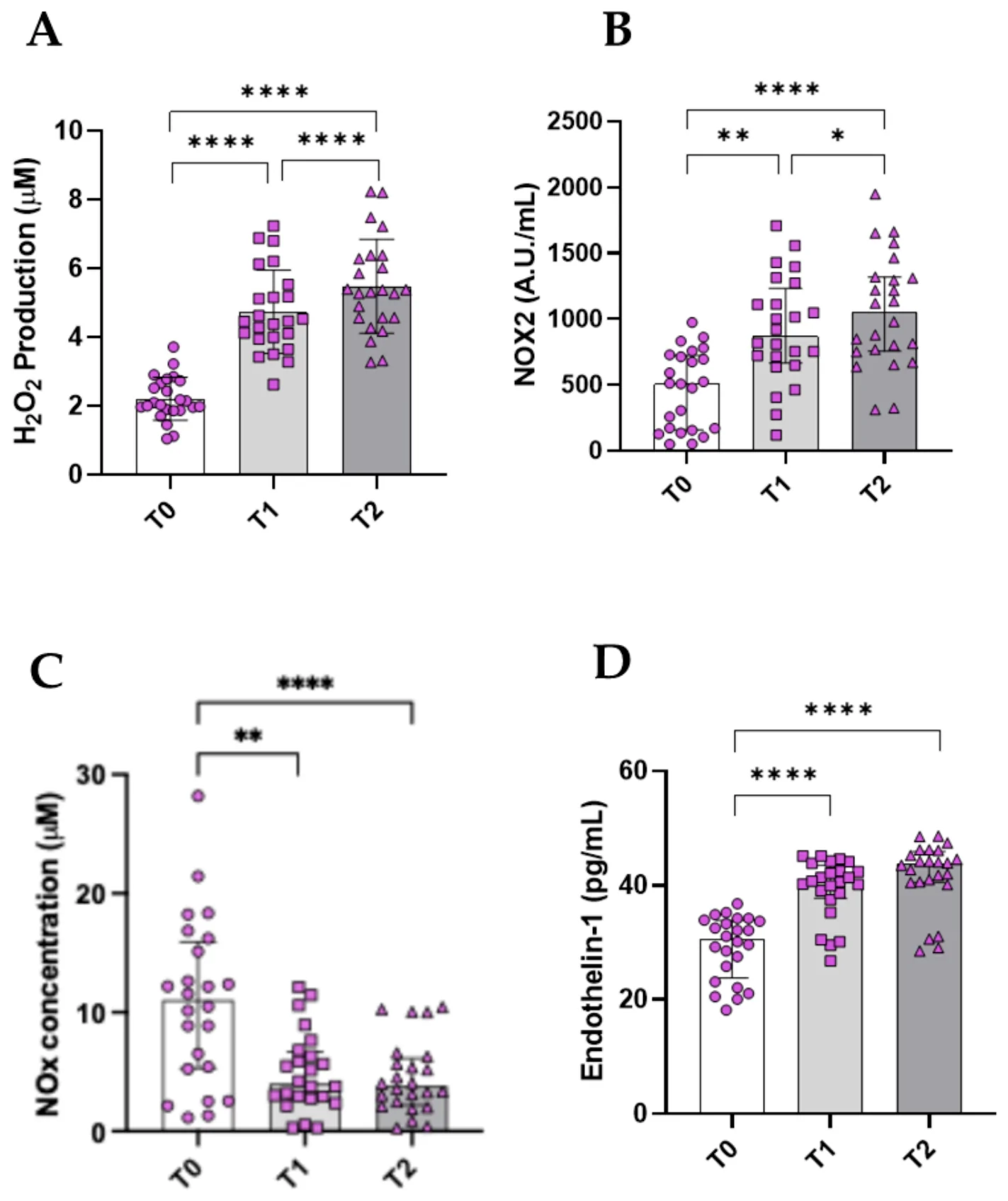
Intensive exercise increases oxidative stress- and endothelial-related alterations in elite athletes. H_2_O_2_ production (**A**), NOX2 (**B**), NOx concentration (**C**), and endothelin-1 levels (**D**) were measured in salivary samples at three time points: pre-season (T0), after one month of training (T1), and following the first half of the competitive season (T2). For H_2_O_2,_ data are expressed as mean ± SD. Statistical differences were assessed using repeated-measures analysis of variance (ANOVA). For NOX2, NOx concentration, and endothelin 1, data are expressed as medians and interquartile ranges (IQRs). Statistical differences were assessed using the Friedman signed-rank test. * *p* < 0.05; ** *p* < 0.01; **** *p* < 0.0001.

The level of H_2_O_2_ in saliva samples was significantly higher in adolescent athletes at T1 compared with T0 and at T2 compared with T0. Moreover, NOX2 levels were significantly higher in adolescent athletes at T1 and at T2 (Figure 1A,B) compared with T0. We also found increased levels of H_2_O_2_ and NOX2 at T2 compared to T1 (Figure 1A,B).

Regarding biomarkers of endothelial impairment, we found that NOx concentration was significantly reduced at T1 compared to T0 and at T2 compared to T0. These changes were accompanied by a significant increase in ET-1 levels at T1 and at T2 compared with T0 (Figure 1C,D).

These findings suggest that sustained training increased oxidative stress and impaired endothelial function. Interestingly, these alterations remained elevated over time (T2), suggesting persistent oxidative stress and endothelial impairment.

### 3.2. In Vitro Study

**Figure 2:**
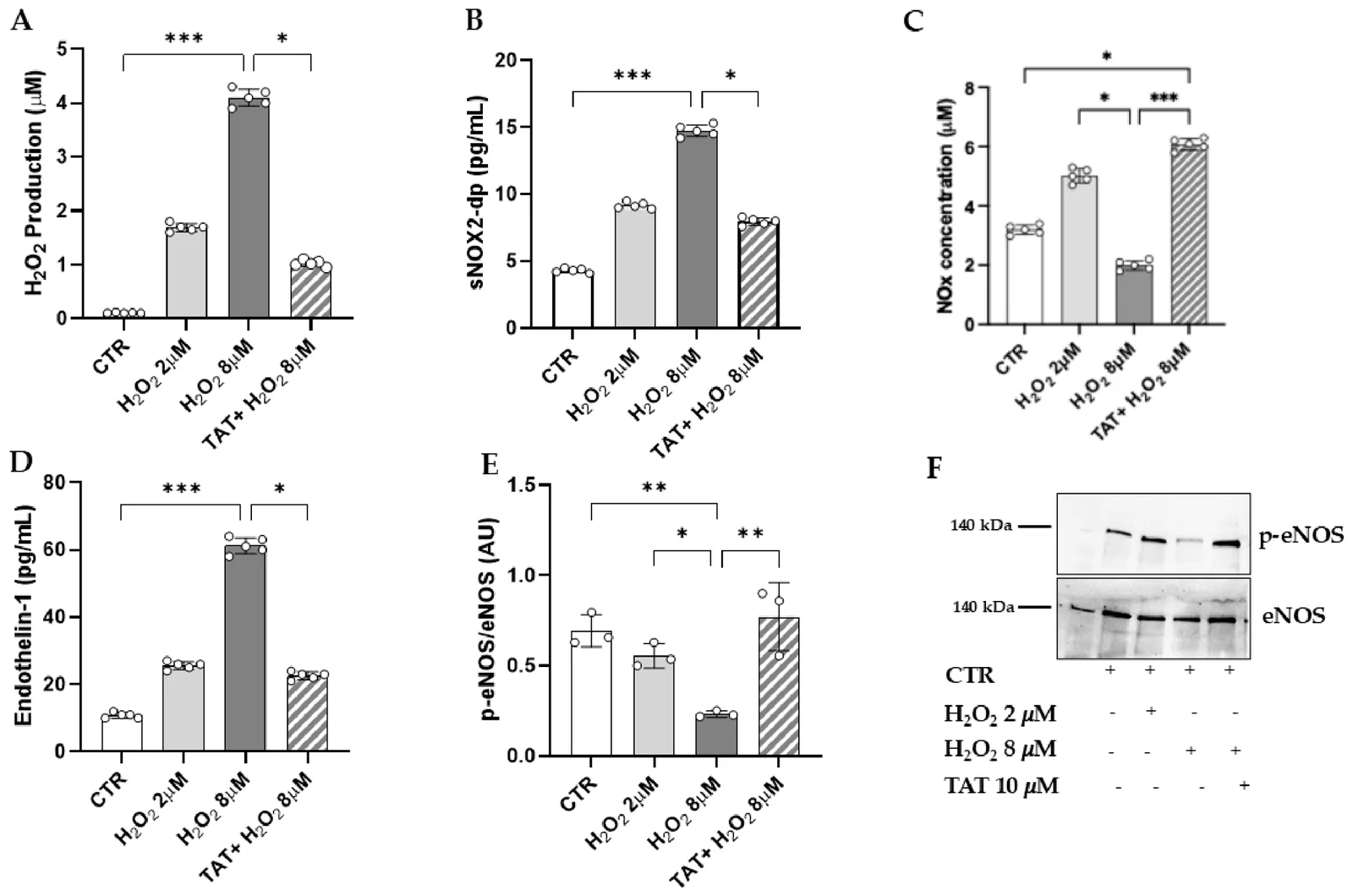
Inhibition of NOX2 reverses H_2_O_2_-induced endothelial-related alterations and oxidative stress in vitro. H_2_O_2_ production (**A**), NOX2 activity (**B**), NOx concentration (**C**), and endothelin-1 levels (**D**) in the supernatants of HUVEC treated with H_2_O_2_ (2 and 8 μM) in the presence or not of NOX2ds-tat as an inhibitor of NOX2. The ratio of phospho-eNOS to total eNOS (**E**) and representative Western immunoblots of phospho-eNOS-S1177 and total eNOS (**F**) in HUVECs treated with H_2_O_2_ (2 and 8 μM) in the presence or not of NOX2ds-tat. Data are represented as mean ± SD of three independent experiments. * *p* < 0.05; ** *p* < 0.01; *** *p* < 0.001. CTR = control; TAT = NOX2ds-tat.

To further substantiate the role of NOX2-mediated oxidative stress in endothelial impairment, we conducted an in vitro study using human endothelial cells. Human umbilical vein endothelial cells (HUVECs) were incubated with increasing concentrations of H_2_O_2_ (0, 2, or 8 μM), at levels comparable to those detected in the saliva samples of adolescent athletes. To investigate the mechanisms underlying endothelial damage, oxidative stress, and endothelial function, these processes were assessed in the supernatant of treated cells by measuring (1) the concentration of soluble NOX2-derived peptide (sNOX2-dp), a marker of NOX2 activation and H_2_O_2_ production; (2) NOx concentration; and (3) ET-1 concentration. Exposure to 8 μM H_2_O_2_ induced a significant increase in H_2_O_2_, sNOX2-dp levels, and ET-1 levels, along with a marked reduction in NOx concentration, indicating increased oxidative stress and impaired endothelial function (Figure 2A–D). Notably, the addition of a specific NOX2 inhibitor (NOX2ds-tat) significantly attenuated oxidative stress and partially restored endothelial function (Figure 2A–D).

When HUVECs were incubated with 8 µM exogenous H_2_O_2_, the measured concentration of extracellular H_2_O_2_ after 3 h reached approximately 4 µM (Figure 2A). This reduction in detectable H_2_O_2_ reflects the active intracellular and cell-surface enzymatic scavenging capacity (via catalase and peroxiredoxins) of endothelial cells. Furthermore, exposure to exogenous H_2_O_2_ triggered intracellular NOX2 activation (ROS-induced ROS release mechanism), as demonstrated by elevated sNOX2-dp levels (Figure 2B). The addition of the specific NOX2 inhibitor NOX2ds-tat abolished this endogenous amplification loop, bringing detectable H_2_O_2_ and sNOX2-dp levels back down to baseline (Figure 2A,B) and significantly restoring endothelial parameters (Figure 2C,D).

To better elucidate the molecular mechanisms underlying these effects, we examined the phosphorylation status of endothelial nitric oxide synthase (eNOS) at Ser1177, a critical regulator of nitric oxide (NO) production and endothelial homeostasis [30]. Our results showed that treatment with 8 μM H_2_O_2_ significantly reduced eNOS phosphorylation, whereas pretreatment with NOX2ds-tat restored phosphorylation levels (Figure 2E,F).

**Figure 3:**
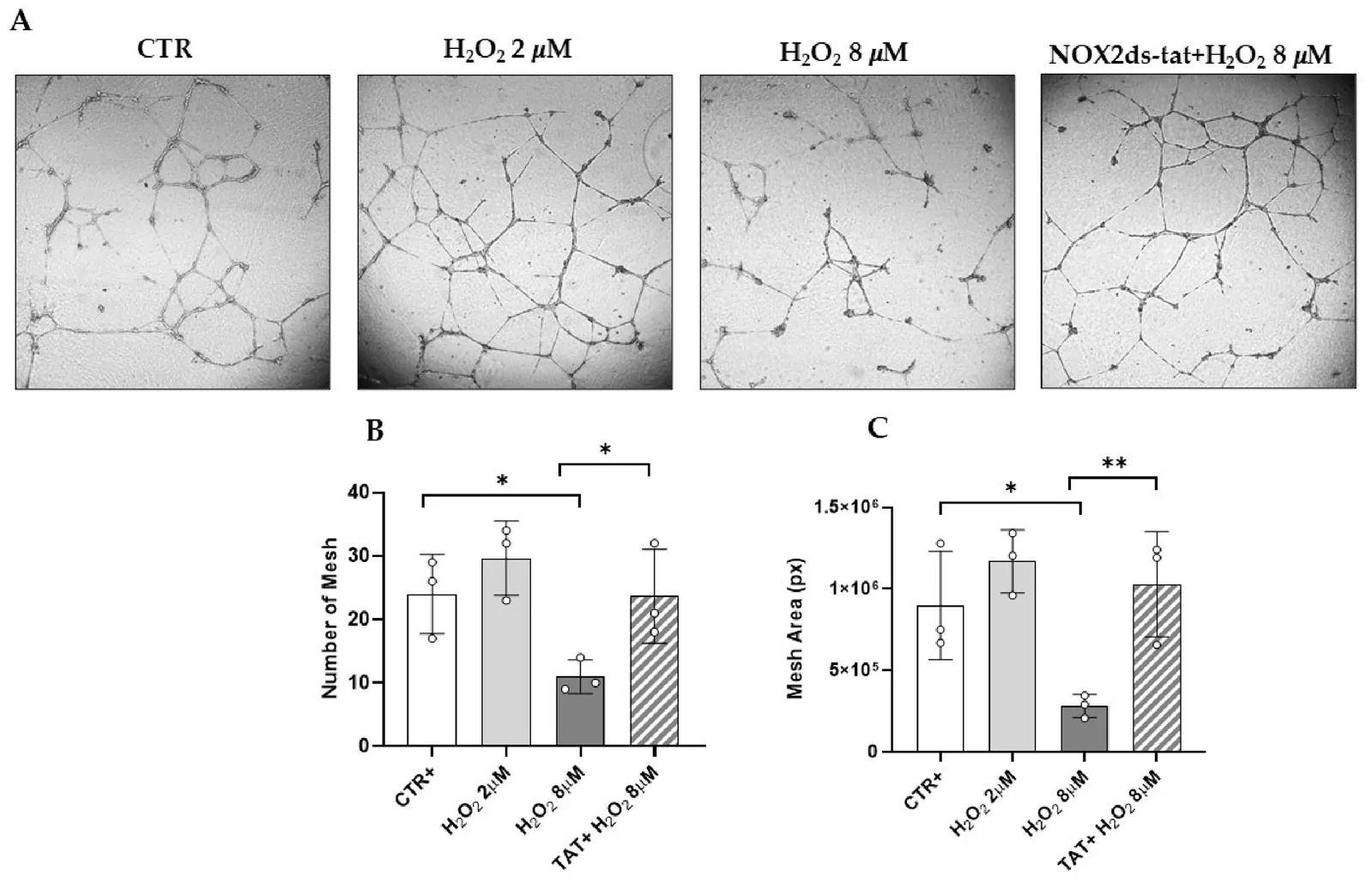
Inhibition of NOX2 restores angiogenesis impaired by H_2_O_2_. Representative images of tube formation in HUVECs cultured on Matrigel treated with H_2_O_2_ (2 and 8 μM) in the presence or not of NOX2ds-tat as an inhibitor of NOX2 activity (**A**). Quantification of (**B**) mesh number and (**C**) mesh area. Data represent the mean ± SD of three independent experiments. * *p* < 0.05; ** *p* < 0.01. CTR = control; TAT = NOX2ds-tat.

Finally, using a Matrigel assay, we assessed the effects of H_2_O_2_ on the angiogenic properties of endothelial cells. H_2_O_2_-treated cells impaired tube formation, reducing mesh number and mesh area (Figure 3A–C). NOX2ds-tat restored angiogenic capacity, significantly increasing both parameters.

## 4. Discussion

The present study investigated the impact of intense training on salivary oxidative stress- and endothelial-related biomarkers in elite adolescent soccer players. Our findings demonstrate that sustained high-intensity training during the competitive season is associated with a modulation of salivary redox- and endothelial-related markers. We observed increased NOX2 levels and H_2_O_2_ levels, along with reduced NOx concentration and elevated endothelin-1 levels. Salivary biomarkers serve as practical, non-invasive surrogate indicators reflecting redox- and endothelial-related signaling rather than direct quantitative measures of systemic or vascular concentrations [31]. Furthermore, because direct functional vascular assessments (e.g., FMD) were not performed, these changes are described as endothelial-related alterations rather than definitive endothelial dysfunction. These findings may reflect the activation of redox-sensitive pathways affecting vascular homeostasis during intensive training.

Regular physical activity is widely recognized for its beneficial effects on cardiovascular health, primarily through improvements in endothelial function and nitric oxide signaling [32]. Moderate exercise promotes physiological adaptations that enhance endothelial nitric oxide synthase (eNOS) activity and increase NOx concentration, thereby supporting structural and functional vascular remodeling and metabolic adaptations in mitochondrial function and glucose/lipid metabolism [32,33]. ROS generated during exercise are essential physiological signaling molecules required for normal arterial tone regulation, acute vascular responses, and structural adaptations [5]. During early ontogenesis and adolescent growth, baseline ROS-dependent signaling is particularly prominent to drive developmental angiogenesis and vascular maturation [17]. However, the relationship between exercise intensity and vascular health follows a complex dose–response pattern.

Structured and moderate exercise provides numerous health benefits, improving antioxidant defenses and adaptive redox signaling, thereby mitigating the adverse effects of oxidative stress [34]. In contrast, strenuous or prolonged exercise may lead to excessive production of ROS in critical tissues like blood and skeletal muscles, which are significant sources of ROS during exercise, resulting in oxidative stress [34].

In this context, our data show that oxidative stress progressively increased during the training season, as evidenced by higher salivary NOX2 and H_2_O_2_ levels at both T1 and T2 compared with pre-season values. Importantly, these changes were accompanied by reduced NOx concentration and increased endothelin-1 levels, suggesting a modulation of endothelial-related signaling pathways.

Although regular exercise generally promotes endothelial adaptations through physiological increases in shear stress and NO signaling [35], strenuous or sustained training may also be associated with an increased oxidative burden [6]. Excessive ROS production can reduce NO bioavailability through oxidative inactivation and interfere with eNOS-dependent signaling. Because NO exerts a tonic inhibitory effect on endothelin-1 (ET-1) synthesis and release, the loss of bioavailable NO triggers a compensatory upregulation of ET-1 expression, driving endothelial activation and vasoconstrictive balance [36].

Excessive ROS production has been shown to reduce nitric oxide bioavailability through direct chemical inactivation and by altering endothelial nitric oxide synthase activity, thereby contributing to endothelial dysfunction [37]. These findings support the concept that sustained oxidative stress may interfere with nitric oxide signaling and impair vascular homeostasis during periods of intense training. It should be noted that the biomarkers analyzed in this study, particularly those measured in saliva, may reflect both local oral and systemic processes. Therefore, they should be interpreted cautiously as indicators of redox- and endothelial-related overall status rather than direct equivalents of circulating vascular concentrations [31].

Notably, the more pronounced changes observed at T2, despite the shorter training volume compared with T1, may be interpreted in the context of a pre-existing chronic state of low-grade inflammation and oxidative stress in these elite adolescent athletes. Such a condition could potentially amplify the biological response to variations in training load, leading to an exaggerated redox imbalance even after relatively shorter periods of intensified exercise.

Among enzymatic sources of ROS, NADPH oxidases represent key contributors to oxidative stress in the cardiovascular system. The NOX family of enzymes is recognized as one of the main sources of ROS in vascular tissues [38]. In particular, the NOX2 isoform has been extensively implicated in mechanisms of endothelial dysfunction and vascular oxidative stress [39]. Previous experimental and clinical evidence indicates that increased NOX2 activation is associated with vascular impairment and increased systemic oxidative stress [40]. Moreover, recent studies in athletes have reported that intense physical activity may enhance NOX2 activation, suggesting that this enzymatic pathway contributes to exercise-induced oxidative stress [11]. Our findings extend these observations to adolescent athletes, a population in which the effects of sustained training loads on vascular redox balance remain poorly characterized.

Similarly, although endothelin-1 is primarily produced by endothelial cells and released in a polarized manner toward vascular smooth muscle, evidence indicates that salivary endothelin-1 levels are not purely independent of the systemic compartment. Previous studies have reported significant correlations between salivary and plasma concentrations of endothelin isoforms, including ET-1, suggesting a partial reflection of circulating endothelin dynamics in saliva [31]. However, this relationship is not based on a direct equilibrium between compartments, and salivary ET-1 likely represents the integrated contribution of systemic endothelial activation and local oral production. Therefore, salivary ET-1 should be interpreted as a marker of endothelial-related signaling rather than a strict quantitative surrogate of plasma levels. The significant associations observed between oxidative stress markers and endothelial impairment indicators further support the hypothesis that NOX2 activation may represent a key mediator linking intense exercise with vascular alterations. Previous research has demonstrated that oxidative stress and endothelial dysfunction are closely interconnected processes that contribute to early vascular impairment and may represent an early step in the development of cardiovascular disease [41].

To further elucidate the mechanisms underlying these in vivo observations, we conducted in vitro experiments using endothelial cells exposed to H_2_O_2_ concentrations comparable to those detected in athletes. In our in vitro experiments, exposing endothelial cells to 8 µM H_2_O_2_ resulted in a lower detectable concentration (4 µM) in the supernatant after incubation. This highlights the strong endogenous antioxidant buffering capacity of endothelial cells, which rapidly metabolize exogenously added peroxide. However, the initial exogenous H_2_O_2_ stimulus acts as a trigger that activates endogenous NOX2 via a feed-forward mechanism known as ROS-induced ROS release. Pretreatment with the specific NOX2 inhibitor NOX2ds-tat blocked this secondary endogenous ROS amplification, confirming that NOX2 activation acts as a key downstream mediator in H_2_O_2_-induced endothelial impairment and eNOS downregulation. These findings indicate that oxidative stress alone is sufficient to reproduce key aspects of the endothelial alterations observed in athletes. Furthermore, pharmacological inhibition of NOX2 significantly attenuated these deleterious effects, demonstrating that NOX2 activation serves as a key downstream mediator in H_2_O_2_-induced endothelial and signaling impairment. A key component of intact endothelial function is a functional eNOS that is in the luminal endothelial cell membrane, producing NO from L-arginine. Phosphorylation at specific sites, primarily S1177, is a post-translational control mechanism activating eNOS and regulating NO production [30]. We found that higher H_2_O_2_ levels resulted in decreased eNOS phosphorylation, reflected by decreased in vitro NOx production and increased ROS production.

In addition to molecular markers of endothelial impairment, our study also demonstrated functional consequences of oxidative stress on endothelial angiogenic capacity. Endothelial cells exposed to elevated H_2_O_2_ concentrations exhibited impaired tube formation, as evidenced by reductions in both mesh number and mesh area in the Matrigel assay. These alterations suggest that excessive oxidative stress may compromise endothelial repair and angiogenic responses, processes that are essential for maintaining vascular integrity.

Adolescence represents a critical developmental period characterized by rapid growth, hormonal changes, and ongoing maturation of the cardiovascular system [42]. During this phase, exposure to excessive training loads may challenge physiological adaptive mechanisms and induce transient vascular imbalance [43].

Although the oxidative and endothelial changes observed in our study may represent an adaptive response to intense training, their persistence over time raises questions regarding potential long-term cardiovascular implications. Our findings, therefore, highlight the importance of monitoring cardiovascular and redox biomarkers in young athletes engaged in high-intensity sports. Non-invasive approaches, such as salivary biomarker assessment, may be a practical strategy for evaluating oxidative stress and endothelial status during training periods. Furthermore, strategies aimed at optimizing training load, recovery periods, and nutritional antioxidant support may help mitigate excessive oxidative stress while preserving the beneficial effects of exercise.

This study has several limitations that should be acknowledged.

(1) The observational design lacked an age-matched sedentary or non-athletic control group, which limits the ability to isolate exercise-specific responses from age-related developmental changes over the season. While recruiting sedentary adolescent controls with matched lifestyles poses ethical and logistical challenges, using athletes as their own internal baseline controls (T0) minimized inter-individual variance.
(2) In vitro mechanistic studies were performed using HUVECs, a primary human umbilical macrovascular cell line. While HUVECs represent a well-established and standardized model for evaluating primary human endothelial responses and redox pathways, neonatal endothelial cells possess distinct metabolic and receptor profiles compared to mature adult micro- or macrovascular endothelial cells. Future studies should confirm these signaling pathways in adult endothelial models.
(3) The sample size was relatively small and limited to male soccer players, which may restrict the generalizability of the findings to other sports or female athletes. Moreover, the absence of a formal power analysis may increase the risk of Type II errors in analyses.
(4) Salivary biomarkers provide a non-invasive method for monitoring oxidative stress, but they may not fully reflect systemic vascular changes and can be influenced by oral metabolism.
(5) A limitation of the present pilot study is the absence of a detailed quantification of training load (including both internal and external load variables) at each time point, which will be addressed in future expanded studies involving a larger and more heterogeneous sample and the support of exercise physiology specialists.

Finally, the observational design of the in vivo component does not allow definitive causal relationships to be established between training load and endothelial alterations. In addition, we did not evaluate cardiovascular clinical parameters such as endothelial function assessed by FMD, or electrocardiographic or echocardiographic parameters.

Despite these limitations, our study provides novel evidence linking intense training in adolescent athletes to NOX2-mediated oxidative stress and endothelial impairment. The combined in vivo and in vitro approaches strengthen the biological plausibility of our findings and support the concept that excessive oxidative stress may represent an early mechanism affecting vascular homeostasis in young athletes.

## 5. Conclusions

In conclusion, the present findings suggest that sustained high-intensity training during the competitive season is associated with a shift toward a pro-oxidative state and concurrent alterations in endothelial-related biomarkers in elite adolescent soccer players. The consistency between the in vivo observations and the in vitro results supports the biological plausibility of NOX2-dependent oxidative stress as a mechanism involved in the vascular response to intensive exercise. While these changes may reflect physiological adaptations to prolonged training, they also underline the importance of carefully balancing training stimuli and recovery during adolescence, a critical period for cardiovascular development. Further longitudinal studies are warranted to determine whether these alterations are fully reversible or may have implications for long-term vascular health.

## Figures and Tables

**Table 1:** Characteristics of participants at baseline (T0).

Variable	Mean ± SD
**Age (years)**	15 ± 0
**Gender (M/F)**	24/0
**Height (cm)**	174 ± 7.6
**Weight (kg)**	62.6 ± 9.7
**BMI**	20.43 ± 2.3
**Systolic blood pressure (mmHg)**	116.9 ± 12.5
**Diastolic blood pressure (mmHg)**	63.96 ± 4.6
**Training volume T1 (hours/week)**	12 ± 1.5
**Training volume T2 (hours/week)**	9 ± 1.2

## Data Availability

The original contributions presented in this study are included in the article. Further inquiries can be directed to the corresponding author.

## Abbreviations

The following abbreviations are used in this manuscript:

CRP	C-Reactive Protein
eNOS	Endothelial Nitric Oxide Synthase
ET-1	Human Endothelin-1
H_2_O_2_	Hydrogen Peroxide
HRP	Horseradish Peroxidase
hs-cTnT	High-Sensitivity Cardiac Troponin T
HUVECs	Human Umbilical Vein Endothelial Cells
IL-6	Interleukin-6
IQR	Interquartile Range
NOX2	NADPH Oxidase Isoform 2
PBS	Phosphate-Buffered Saline
ROS	Reactive Oxygen Species
SD	Standard Deviation
sNOX2-dp	NOX2-Derived Peptide
TMB	3,3′,5,5′-Tetramethylbenzidine
